# Case Report: Idiopathic Traumatic Neuroma of the Gallbladder Without Previous Surgery

**DOI:** 10.3389/fsurg.2022.851205

**Published:** 2022-06-23

**Authors:** Tianyu Lin, Abdul Saad Bissessur, Yingjie Zhu, Teruko Fukuyama, Guoping Ding, Liping Cao

**Affiliations:** ^1^ Department of Hepatobiliary and Pancreatic Surgery, Sir Run Run Shaw Hospital, School of Medicine, Zhejiang University, China; ^2^ Department of Surgical Oncology, Sir Run Run Shaw Hospital, School of Medicine, Zhejiang University, China; ^3^ School of Medicine, Zhejiang University, Hangzhou, China

**Keywords:** traumatic neuroma, gallbladder neoplasm, idiopathic, cholangiocarcinoma, case report

## Abstract

Traumatic neuroma mostly results from nerve injury caused by surgery or trauma. Traumatic neuroma of the gallbladder without prior abdominal surgery is extremely rare, and we termed it “idiopathic traumatic neuroma of the gallbladder.” Due to its rarity and a lack of specific clinical and radiological features, it is most commonly misdiagnosed. In our case, the patient was admitted to our hospital for cholangiocarcinoma. Repeated abdominal contrast-enhanced computed tomography scans preoperatively indicated hilar cholangiocarcinoma. Due to insufficient future liver remnant, we planned preoperative percutaneous transhepatic cholangiodrainage and percutaneous transhepatic portal vein embolization based on multidisciplinary team consultation. The patient was then admitted 1 month later for surgery. We performed a laparoscopic cholecystectomy and an extensive laparoscopic right hepatectomy as gallbladder carcinoma was strongly suspected intraoperatively. However, the final diagnosis was traumatic neuroma of the gallbladder confirmed by pathological examination. Traumatic neuroma of the gallbladder is very rare, and we hope to provide some references for diagnosis by reporting our case and reviewing the literature on this topic so that extensive treatment can be avoided, thus improving patients’ quality of life. To the best of our knowledge, this is the first reported case of traumatic neuroma without prior surgery in the English literature since 1996.

## Introduction

Neuroma arising in the biliary system is a very rare benign lesion and can be classified into two types: primary and traumatic neuroma (1). Pathologies, such as infection, inflammation, trauma, gallstones, or surgery, involving the biliary tree can lead to traumatic neuroma (2). Traumatic neuroma is a tumor-like proliferation formed at the proximal end of damaged nerves after surgery or trauma, usually presenting as a firm, oval, slowly growing, and painful nodule (3). Traumatic neuroma, also known as amputation neuroma, arising in the gallbladder without prior surgery and cholelithiasis is extremely rare (4).

Diagnosis of a gallbladder neuroma is ascertained based on pathological findings of spindle-shaped cells and immunohistochemistry findings of the S-100 protein of postoperative or biopsy samples (5). Traumatic neuroma usually follows an asymptomatic course (6). Surgical excision remains the mainstay treatment (3). However, traumatic neuroma frequently mimics cholangiocarcinoma and extensive radical surgery is performed (7).

Due to the lack of specific clinical symptoms and imaging findings, it is most commonly misdiagnosed as malignancy. Herein, we report one case of an idiopathic traumatic neuroma of the gallbladder treated at our hospital and preoperatively misdiagnosed as cholangiocarcinoma.

## Case Presentation

A 60-year-old woman presented at our hospital with abnormal liver function tests discovered 2 weeks earlier on physical examination. Enhanced abdominal computed tomography (CT) scans revealed a space-occupying lesion at the hepatic hilum that was presumably considered hilar cholangiocarcinoma. The patient was admitted to our hospital on February 21, 2019. The patient denied any prior abdominal surgery or trauma. No abnormalities were found on physical examination.

Laboratory investigations included liver function enzymes and tumor markers: alanine aminotransferase (ALT) 187 U/L (normal: 7–55 U/L), aspartate aminotransferase (AST) 111 U/L (normal: 8–48 U/L), alkaline phosphatase (ALP) 737 U/L (normal: 40–129 U/L), gamma-glutamyltransferase (γ-GT) 1,074 U/L (normal: 8–61 U/L), total bilirubin (TBil) 28.5 μmol/L (normal: 4–19 μmol/L), direct bilirubin (DBil) 13.7 μmol/L (normal: 0–5 μmol/L), albumin 44.5 g/L (normal: 30–50 g/L), CA19-9 50.01 U/ml (normal: 0–37 U/ml), CA125 12.18 U/ml (normal: 0–35 U/ml), alpha fetoprotein 8.63 ng/ml (normal: <20 ng/mL), and CEA 1.93 ng/ml (normal: 0–2.5 ng/mL).

**Figure 1:**
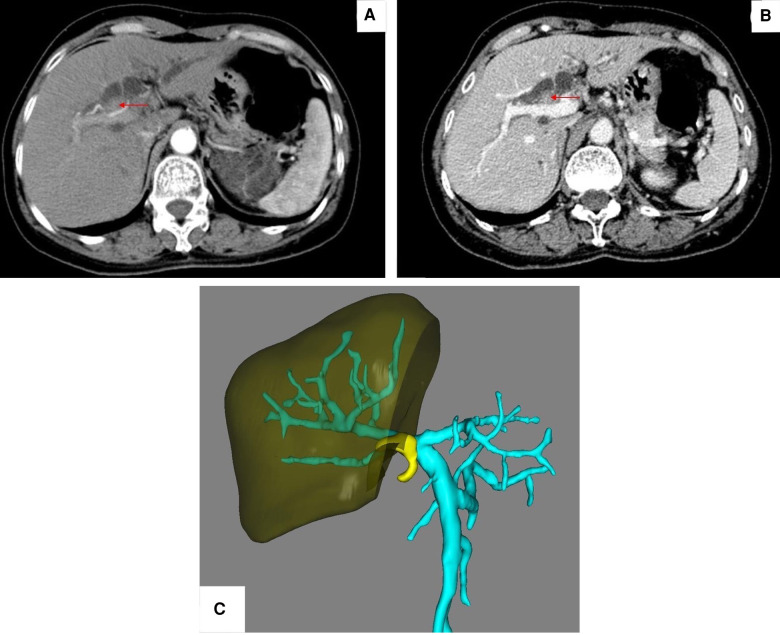
(**A**) CT revealed a 10 × 17 mm space-occupying lesion (red arrow) in the hepatic hilum with an unclear boundary. (**B**) Tumor (red arrow) showed obvious enhancement. (**C**) Postoperative left residual volume was estimated to be only 27.6%.

Abdominal enhanced CT scan (Figure 1A) revealed a 10 × 17 mm mass in the hepatic hilum with an unclear boundary. In the arterial phase (Figure 1B), the mass showed obvious enhancement. The intrahepatic bile duct showed obvious dilation, and the mass surrounded the cystic duct. The wall of the gallbladder appeared thickened, but there was no significant enlargement. No enlarged lymph nodes were noted in the retroperitoneum. Furthermore, 3D reconstruction revealed a space-occupying lesion at the hilar bile duct, invading the right branch of the portal vein. Furthermore, the postoperative left residual liver volume (RLV) was estimated to be only 27.6% (Figure 1C).

After a multidisciplinary team (MDT) discussion, the location of the tumor was considered unordinary, and, since a biopsy would have been highly risky, the pathological nature of the tumor was difficult to establish preoperatively. Thus, preoperative diagnosis was considered to be hilar cholangiocarcinoma based on enhanced CT scans. However, direct surgical intervention (right hepatectomy) would be inappropriate due to insufficient residual liver volume. The patient thus underwent preoperative percutaneous transhepatic cholangiodrainage (PTCD) on March 1, 2019, followed by percutaneous transhepatic portal vein embolization (PVE) on March 4, 2019, to improve residual liver volume.

**Figure 2:**
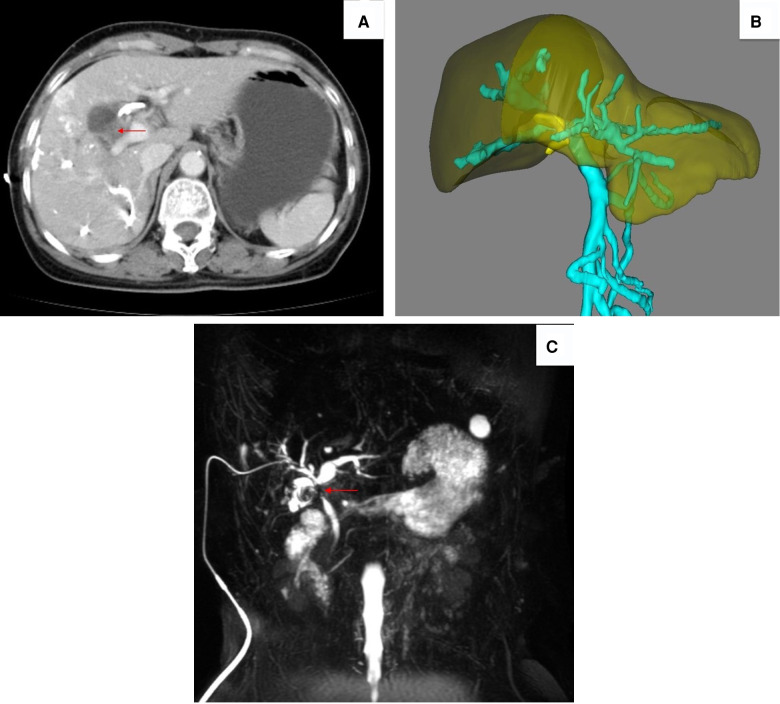
(**A**) Abdominal CT 1 month later revealing an improvement in hepatic duct dilation (the red arrow designates the tumor). (**B**) Residual liver volume (RLV) increased up to 55.57%. (**C**) MRCP indicating a space-occupying lesion (red arrow) at the porta hepatitis, involving the cystic duct.

One month later, the patient was admitted again for surgery. Preoperative laboratory investigations revealed elevated liver function enzymes (ALT 131 U/L, AST 256 U/L, ALP 188 U/L, γ-GT 209 U/L), while bilirubin and tumor markers were all within the normal range. A contrast-enhanced CT (Figure 2A) of the abdomen revealed improvement in the dilation of the hepatic duct, while the volume of the left hepatic liver had increased by 55.57% (Figure 2B). Magnetic resonance cholangiopancreatography (MRCP) (Figure 2C) indicated an irregular shadow at the porta hepatitis with a vague boundary and involving the cystic duct. The gallbladder was not enlarged, but the walls were significantly thickened.

**Figure 3:**
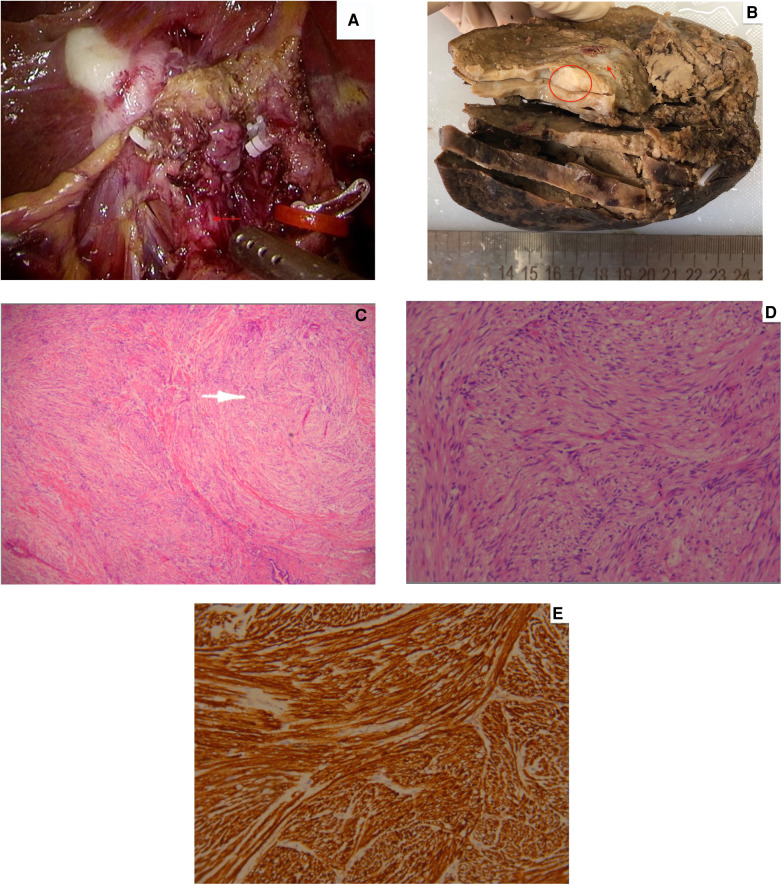
(**A**) Intraoperative findings revealing an atrophied gallbladder, with a hardened texture (the red arrow points out the tumor). (**B**) Grayish-white nodule (pointed by the red arrow) seen at the neck of the gallbladder (represented by the red circle). (**C**) Low-magnification and (**D**) high-magnification pathological analysis showing spindle cells. (**E**) Immunohistochemical positivity of S-100.

On April 11, 2019, the patient underwent surgery. Intraoperatively, the gallbladder was severely attached to the liver. The gallbladder had atrophied with a hardened texture (Figure 3A). The tumor invaded the hilar bile duct and the right branch of the portal vein. Lymph node enlargement at the hepatoduodenal ligament was observed. Based on the intraoperative findings, gallbladder carcinoma was considered. The surgical intervention included laparoscopic cholecystectomy, laparoscopic right extended hepatectomy, and laparoscopic hepatoduodenal ligament lymph node dissection. A cholangiojejunostomy was then performed. Intraoperative freezing biopsy showed no cancer cells at the level of the resected left bile duct and cystic duct.

Postoperative pathological examination revealed that the mucosa of the gallbladder was grayish-white with a hard texture. Fibrous tissue hyperplasia of the gallbladder wall with hyaline degeneration was seen together with chronic interstitial inflammatory cell infiltration and focal multinucleated giant cell aggregation. A nodule, 1.4 × 1.0 cm in size, with a grayish-whitcross-sectionon (Figure 3B), was seen at the neck of the gallbladder. Pathological analysis and immunohistochemistry validated the diagnosis of traumatic neuroma through spindle cells (Figure 3C) and S-100 positivity (Figure 3D). Four resected lymph nodes showed reactive hyperplasia. The final validated diagnosis was traumatic neuroma of the gallbladder, chronic cholecystitis, fibrosis of the gallbladder wall, hyaline degeneration, and gallstones. The patient recovered well after the operation, without complications, and was discharged from the hospital. There was no obvious discomfort in the postoperative follow-up.

## Discussion

Benign neoplasms of the biliary system are most commonly adenomas or papillomas and account for only 6% of all biliary tumors (8). However, the actual incidence of gallbladder neuroma remains uncertain as the patients most commonly follow an asymptomatic course. According to an autopsy study of post-cholecystectomy patients, 11 out of 40 patients had histological findings consistent with traumatic neuromas, revealing that 25% of cholecystectomy patients develop traumatic neuromas after surgery (9).

Traumatic neuroma is a non-neoplastic overgrowth of tissues and disorderly proliferation of nerve fibers and most frequently arises after radical neck dissection, orthopedic or limb amputation surgeries (3, 10). Traumatic neuroma of the biliary system emerges from the sympathetic and parasympathetic nerve endings surrounding the bile ducts and gallbladder (2). As the common bile duct is a rich innervated area (11), traumatic neuroma commonly occurs after cholecystectomy or even bile duct exploration (12). The tumor commonly arises at the cystic duct stump or remnant after cholecystectomy (13). Traumatic neuroma can occur as early as 1 month and as late as 56 years post-cholecystectomy (14). However, traumatic neuroma arising in the gallbladder is a rare occurrence (15). Moreover, due to sustained injuries, traumatic neuroma can occur after orthotopic liver transplantation (16, 17). Rarely, traumatic neuroma might arise in the gallbladder post-vagotomy (18).

Even though most patients follow an asymptomatic course, some patients present with abnormal liver function, jaundice, pruritus, abdominal pain, weight loss, abnormal urine color, or clay-colored stools (1, 7). Our patient presented with abnormal liver function tests. Surgical excision remains the mainstay treatment of traumatic neuroma, and there is no response to chemotherapy or radiotherapy (3). Due to its rarity and the limitations of specific imaging findings, traumatic neuroma of the biliary tract is most commonly misdiagnosed as cholangiocarcinoma (7), as happened in our case. Neuroma is composed of disordered nerve fibers and its signal density is the same as soft tissue and the pancreatic head, thus making it difficult to identify by CT and MRI examinations (19). Just as in our case, the elevation of tumor marker CA19-9 (7) and liver function enzymes (1) makes correct preoperative diagnosis a real challenge.

A case report by Shimura et al. (6) proved that intraductal ultrasonography (IDUS) could demarcate traumatic neuroma from other malignancies as it provided accurate details. The IDUS showed that the lesion demonstrated a smooth homogeneously hypoechoic mass with a distinct margin and was able to determine whether the surrounding structures such as the portal vein, hepatic artery, and pancreas were invaded. These characteristics pointed toward traumatic neuroma, which meant that extensive surgery could be avoided. Furthermore, two post-cholecystectomy cases of traumatic neuroma were diagnosed by biopsies through the combined use of a cholangioscopy and an endoscopic ultrasound (5, 14). In our case, however, the tumor was close to the hepatic hilum, making a preoperative biopsy difficult.

In this case, the traumatic neuroma was located in the gallbladder. Erratically, our patient had no prior history of abdominal surgery, gallstones, or polyps. Since there are no typical causes, such as surgery and physical trauma, we delineate such a finding as “idiopathic traumatic neuroma of the gallbladder.” PubMed, Embase, CNKI, and other websites were thoroughly searched for relevant publications, and only four cases of traumatic neuroma without previous surgery have been reported in the last 60 years (Table 1). All the patients were elderly males, of which two patients presented with abdominal pain and gallstones (4, 20–22). In contrast, our patient was an elderly female presenting with abnormal liver function enzymes. In one of the cases (22), reported in the Japanese literature, physical stimuli to surrounding nerves from a pedunculated polyp were speculated to be the causative factor of the neuroma in the gallbladder.

**Table 1:** Case reports of idiopathic traumatic neuroma of gallbladder published in the past 60 years.

Study	Age	Sex	Symptoms	Gallstones	Pre-operative diagnosis	Surgical scope
Janes et al. (4)	66	M	Epigastric pain, jaundice	Present	Gallstones	LC converted to OC, repair of fistula b/w the gallbladder and colon
Matsuoka et al. (20)	74	M	None (incidental finding)	Absent	Cholangiocarcinoma	LC converted to OC, partial liver resection
Peison et al. (21)	88	M	RUQ abdominal pain, abdominal distension, loss of appetite	Present	Gallstones	Cholecystectomy
Yoshida et al. (22)	68	M	None (incidental finding)	Absent	Cholangiocarcinoma	Cholecystectomy + hepatic bed resection

*M, male; LC, laparoscopic cholecystectomy; OC, open cholecystectomy; b/w, between; RUQ, right upper quadrant.*

It is worth mentioning that long-term chronic inflammation was confirmed pathologically in our case and the above-mentioned cases. Physical trauma from surgery or gallstones or polyps may not be the only triggering factors for traumatic neuroma formation in the gallbladder; chronic inflammation may also spawn traumatic neuroma (23). Long-term continuous inflammation and destruction are required to cause a large number of abnormal proliferation of nerves in the gallbladder wall. Hence, chronic cholecystitis could be the inductive factor in our case, thus the reason why we delineate it as “idiopathic traumatic neuroma of the gallbladder.” The rarity of traumatic neuroma could be accounted for by the fact that the nerves near the mucosal surface are too thin to produce colossal proliferation enough to lead to the formation of a neuroma (20). Moreover, chronic inflammation can also cause severe local adhesion, gallbladder rupture, and fistula of the gallbladder with the colon, thereby forming an illusion similar to tumor infiltration, resulting in incorrect intraoperative diagnosis and expanding the scope of surgery. In this case, the gallbladder closely adhered to surrounding tissue.

In patients presenting with a history of abdominal surgery such as a cholecystectomy or liver transplant, traumatic neuroma should be included in the differential diagnosis, especially if the tumor arises at the remnant stump of the bile duct. However, even without prior surgery, inflammation triggered by gallstones or polyps or idiopathic inflammation can be an etiology of traumatic neuroma. While imaging modalities are not specific, liver function enzymes and tumor markers are elevated, and an intraoperative frozen biopsy should be incorporated to avoid extensive radical surgery, thus improving patients’ quality of life. Although idiopathic traumatic neuroma of the gallbladder is very rare, it should be included in the differential diagnosis in patients diagnosed with hilar cholangiocarcinoma.

## Data Availability

The original contributions presented in the study are included in the article/Supplementary Material, further inquiries can be directed to the corresponding author/s.
